# Diabetic ketoacidosis as first presentation of latent autoimmune diabetes in adults in a patient with hashitoxicosis as first presentation of Hashimoto’s thyroiditis: a case report

**DOI:** 10.1186/s13256-022-03523-3

**Published:** 2022-08-03

**Authors:** Maria Xenou, Ioannis Zoupas, Dimitrios Lygnos, Evangelos Fousteris

**Affiliations:** 1 Mediterranean Diabetes and Obesity Clinics (MEDOC), Athens, Santorini Greece; 2 Medical School, National and Kapodistrian University of Athens, Athens, Greece

**Keywords:** LADA, Diabetic ketoacidosis, Hashimoto’s disease, Hashitoxicosis, Case report

## Abstract

**Background:**

Latent autoimmune diabetes in adults is an infrequent form of autoimmune diabetes mellitus, while Hashimoto’s thyroiditis, the most common thyroid disease in adults, rarely manifests as thyrotoxicosis. The concurrent initial presentation of these two autoimmune disorders is extremely rare.

**Case presentation:**

A 29-year-old male of Albanian descent presented after being hospitalized owing to diabetic ketoacidosis. The diagnosis of type 1 diabetes mellitus was placed, and intensified insulin therapy was initiated. Medical history was not of significance except a 5 kg weight loss within 2 months. The patient presented with recurrent episodes of hypoglycemia, and the doses of preprandial and basal insulin were reduced. The differential diagnosis included type 1 diabetes mellitus “honeymoon” period or another type of diabetes mellitus. His serological tests only revealed positive autoantibodies against glutamic acid decarboxylase 65 and C-peptide. The diagnosis leaned toward latent autoimmune diabetes in adults, and the therapeutic approach involved cessation of preprandial insulin therapy, regulation, and subsequent discontinuation of basal insulin and introduction of metformin. Two years later, basal insulin was reintroduced along with a glucagon-like peptide-receptor agonist and metformin. Further physical examination during the initial visit disclosed upper limb tremor, lid lag, excessive sweating, increased sensitivity to heat, and tachycardia. Laboratory tests were indicative of hashitoxicosis (suppressed level of thyroid-stimulating hormone, high levels of total and free thyroid hormones, positive anti-thyroglobulin and anti-thyroid peroxidase, and negative anti-thyroid-stimulating hormone receptor). Thyroid-stimulating hormone level was spontaneously restored, but an increase was observed during follow-up. Levothyroxine was administrated for 2 years until the patient had normal thyroid function.

**Conclusions:**

The prevalence of thyroid autoantibodies in patients with latent autoimmune diabetes in adults ranges from 20% to 30%. This correlation can be attributed to genetic involvement as well as disorders of immune tolerance to autoantigens. Hence, this report gives prominence to the holistic approach and consideration of comorbidities in patients with diabetes mellitus.

## Background

Type I diabetes mellitus and latent autoimmune diabetes of the adults are forms of autoimmune diabetes mellitus that occur after destruction of the pancreatic β-cells, which leads to insulin depletion [1]. Latent autoimmune diabetes in adults (LADA) is rarely observed: 2–12% of newly diagnosed diabetes mellitus (DM) in adults [2]. These patients present diabetes at adulthood and usually do not require insulin therapy immediately after their diagnosis, as they present primarily with symptoms and signs of type 2 DM (T2DM) [3, 4]. Diabetic ketoacidosis (DKA) most commonly appears as the first clinical presentation of T1DM [5], but as the first manifestation of LADA it is extremely rare, and very few cases have been described to date [6–9].

Hashimoto’s disease (HD) is an inflammatory autoimmune disease of the thyroid gland, which clinically manifests most commonly as goiter with or without hypothyroidism [10]. Hashitoxicosis is characterized by the pathological features of HD accompanied by an initial hyperthyroidism burst that later recedes. This phenomenon happens owing to the uncontrolled release of thyroid hormones during the active phase of the disease [11]. According to previous studies [12, 13], hashitoxicosis occurs in about 4.47–11.67% of both adult and children patients with HD.

We present a patient with LADA and Hashimoto’s disease who experienced both DKA, as first manifestation of LADA, and hashitoxicosis on HD. As far as we know, similar cases have been reported only under the influence of administered drugs or coexisting disease [14].

## Case report

A 29-year-old male Albanian patient presented to our clinic owing to recurrent episodes of hypoglycemia. He referred to an incident of DKA that led to hospitalization abroad 20 days ago. The discharge report from the hospital referred to the DKA incident, but mentioned no precipitating factors. The patient was newly diagnosed with T1DM at this time, and intensified treatment with insulin at home was commenced (25 units of basal and 4–8–6 units of preprandial insulin per day). He had been going through stressful times, as he had recently received news of an upcoming fatherhood. He has a body mass index (BMI) of 21.6 kg/m^2^ and an unremarkable personal and family health record. He mentioned a 5 kg weight loss during the past 2 months. Laboratory tests exhibited glycosylated hemoglobin (HbA1c) at 8.1%, C-peptide at 1.8 ng/ml (normal range 0.5–2 ng/ml), and fasting plasma glucose (FPG) at 120 mg/dl. Antibody tests for anti-tyrosine phosphatase-related islet antigen 2 (anti-IA2) were upper normal (7.3 IU/ml, normal range 0.0–7.4 IU/ml), positive for anti-GAD65 (5.5 IU/ml, normal range 0–5 IU/ml), and negative for anti-islet cell antibodies (ICA) and anti-insulin antibodies (IAA). Initial management included significant downtitration of preprandial insulin. Basal insulin dosage was decreased at about 20 IU daily (initial dosage was 0.2 times the patient’s body weight: 0.2 × 70 kg = 14 units of degludec). Differential diagnosis consisted of T1DM “honeymoon” period and possible misdiagnosis at the hospital. Prolonged T1DM honeymoon periods have been described in the world literature, but the age of the patient, along with hypoglycemic incidents after insulin administration, positive anti-GAD65, C-peptide levels, and positive anti-IA2 and negative T1DM antibodies (anti-ICA, anti-insulin), suggested a type of diabetes other than the initially diagnosed T1DM, most likely LADA, according to the 2020 American Diabetes Association criteria [4]. On a follow-up visit, considering the persistent laboratory and clinical findings, we discontinued the administration of preprandial insulin, regulated the basal insulin dosage, and enhanced treatment with metformin. One month later, under treatment with 2 g of metformin daily and additional sitagliptin, basal insulin dosage decreased at about 8 units per day and was subsequently halted. Two years later, basal insulin needed to be reintroduced, along with metformin and a glucagon-like peptide-1 receptor agonist (GLP-1 RA). Insulin requirement after at least 6 months from original diagnosis confirmed our previous hypothesis of LADA, which first manifested with DKA. On a 6-year follow-up, according to laboratory findings and glucose measurements, diabetes progressed to T1DM and basal bolus insulin treatment (multidose insulin, MDI) with detemir and lispro was initiated. The patient remains adherent to the regimen, and it has not been modified since.

In addition, at the initial visit, the patient complained of excessive sweating and increased sensitivity to heat. A complete physical examination (PE) revealed upper limb tremor, lid lag, palpable thyroid gland, and heart rate of 110 beats per minute. Thyroid-stimulating hormone (TSH) levels were lower than 0.004 mIU/L (normal range 0.5–5 μIU/ml), total triiodothyronine (T3) at 2.19 nmol/L (normal range 0.9–2.8 nmol/L), total thyroxine (T4) at 14.60 μg/dl (normal range 5–12 μg/dl), free T3 (fT3) at 4.93 pmol/L (normal range 2–7 pmol/L), and free T4 (fT4) at 1.65 ng/dl (normal range 0.8–1.8 ng/dl). There were positive anti-thyroglobulin (anti-TG) (174 IU/ml, normal range < 116 IU/ml) and thyroid peroxidase antibodies (anti-TPO) (245 IU/ml, normal range < 16 IU/ml), while anti-TSH-R antibody was negative. An ultrasound scan of the thyroid gland was not indicative of pathology. Seven days later, a thyroid panel was conducted: TSH, 3.14 μIU/ml; T3, 1.57 nmol/L; T4, 87.19 μg/dl; fT3, 5.60 pmol/L; fT4, 15.26 ng/dl. TSH values decreased spontaneously with no treatment admitted. High levels of total and free thyroid hormones and presence of elevated anti-thyroid antibodies with negative anti-TSH-R navigated the diagnosis toward hashitoxicosis complicating Hashimoto’s disease and made Graves’ disease less likely. One month later, TSH was measured in a laboratory and found to be higher than normal (7.29 mIU/L), so levothyroxine treatment with maximal dosage of 88 μg was decided. On a 2-year follow-up, thyroxine was withdrawn as Hashimoto’s thyroiditis retreated, and the patient has remained euthyroid (TSH and thyroid hormone levels close to normal on blood test). On a 6-year follow-up, there were no identifiable changes in the patient’s thyroid state.

## Timetable

**Table:** 

5 May 2013	Hospitalization due to DKA and initiation of basal bolus insulin therapy (25 units of basal and 4–8–6 units of preprandial insulin per day)
25 May 2013	Episodes of hypoglycemia, downtitration of basal at approximately 20 units of glargine, and preprandial insulin at 4 units per meal. Clinical and lab signs of hashitoxicosis
13 June 2013	Hypoglycemia persists, preprandial insulin is discontinued, basal insulin is decreased, and the therapeutic scheme is enhanced with a combination of metformin and a DPP4 inhibitor. TSH levels are restored with no need for treatment
27 June 2013	Basal insulin is decreased at 8 units daily and is finally discontinued. Treatment comprises metformin and sitagliptin. TSH levels are found to be higher than normal, and levothyroxine is added
3 July 2015	Basal insulin is reintroduced along with metformin and a GLP-1 agonist. Levothyroxine is withdrawn, and the patient remained euthyroid
14 April 2019	Laboratory and clinical findings are consistent with T1DM. Basal bolus insulin with MDI is initiated. Patient’s glycemic and thyroid conditions remain well regulated ever since

## Discussion and conclusions

LADA, or type 1.5 DM, is the most common form of adult-onset autoimmune diabetes [2]. On the basis of the Immunology of Diabetes Society, LADA is diagnosed with the following criteria: (1) adult-onset age (> 30 years), (2) presence of any islet cell autoantibody, (3) absence of insulin requirement for at least 6 months after diagnosis [4]. LADA is characterized by a genetic mixture of T1DM and T2DM [15], sharing same susceptibility markers with each (HLA, *INS*, *VNTR*, *PTPN22*, and *TCF7L2*, respectively) [16]. This genetic combination could justify serological diversity [16]. Mainly four types of islet autoantibodies are present in patients with LADA: anti-GAD65, IAA, anti-IA2, and zinc transporter 8 antibody (ZnT8A), with anti-GAD65 being the most sensitive marker [17]. Patients with LADA are more likely to be positive for only one autoantibody type. In the Action LADA study, 68.6% of patients were positive only for anti-GAD65, 5% for anti-IA2, and 2.3% for ZnT8A, in comparison with 24.1% developing at least two types of autoantibodies [18]. Fourlanos *et al*. proposed anti-GAD65 testing for patients with two or more of the following criteria: (1) age < 50 years, (2) acute symptoms, (3) BMI < 25 kg/m^2^, and (4) personal or family history of autoimmune disease [19].

Though anti-GAD65 is the most predominant autoantibody in LADA, other islet autoantibodies, namely anti-IA2, ZnT8A, and IAA, are valuable for the LADA diagnosis as well as the prediction of β-cell failure and multiple organ autoimmunity. This recommendation is based on the fact that, besides serological heterogeneity, LADA is also characterized by clinical heterogeneity [16]. In total, 78.5% of patients with LADA have high titer (> 200 World Health Organization (WHO) units) of anti-GAD antibodies [18]. Higher anti-GAD65 titer is associated with more of a “T1DM” clinical image, including accelerated β-cell loss [20], lower BMI, younger age, increased risk of ketosis (DKA), and earlier need for insulin treatment [18, 21, 22], whereas lower levels are commonly correlated with “T2DM” characteristics [22]. Furthermore, being positive for both anti-GAD65 and anti-IA2, as in our patient, increases the possibility of prematurely developing insulin dependence [23] and having a phenotype more similar to T1DM.

As a result, in everyday practice, LADA is often misdiagnosed as T2DM or T1DM, as occurred in our patient. Justifying the initial approach, insulin was the most appropriate therapeutic choice in a patient with weight loss and DKA [24]. DKA is a medical emergency and is characterized by the accumulation of ketoacids in the circulation due to excessive liver ketogenesis occurring because of insulin deficiency. It manifests with hyperglycemia, as a result of partial or complete depletion of insulin and high concentration of hyperglycemic agents [5]. The most common cause of DKA is the onset of T1DM, but several other factors may trigger this clinical demonstration [5, 25, 26]. Patients with LADA rarely present with acute onset or diabetic ketoacidosis (DKA) at diagnosis, as β-cell destruction, compared with T1DM, is significantly delayed [27].

Hashimoto’s disease was first described by Dr. Hakaru Hashimoto in 1912 and has the highest incidence among all other autoimmune diseases. Women have eight times higher risk of being affected compared with men [10, 28]. The typical histopathological trait is lymphocytic infiltration of the thyroid gland. Thyroid peroxidase antibodies (anti-TPO) are present in 95% of patients, and 60–80% are positive for TG antibodies [10]. Clinical features include both local and multisystemic symptoms [10], and different variants have been described. A remarkable variant is hashitoxicosis and affects less than 5% of patients with HD [29]. Hashitoxicosis is a transient period of hyperthyroidism, caused by inflammatory-mediated destruction of thyroid follicles, which leads to massive release of preformed thyroid hormones [11, 30]. Similarly to our patient, Nabhan *et al*. identified no risk factors for the development of hashitoxicosis [13] in patients with HD. Hashitoxicosis combines the microscopic appearance of HD with the clinical image of Graves’ disease [10], thus including glandular enlargement, weight loss, tremor, and heat intolerance, confirming our patient’s symptoms [31, 32]. Contrary to our patient, though, ultrasound findings usually reveal hypoechogenicity [10]. Ultimately, hashitoxicosis falls into euthyroidism or hypothyroidism [33].

There is adequate evidence that autoimmune diseases interact with each other when it comes to onset, clinical manifestations, and frequency. Many studies have proved the strong connection between autoimmune DM and autoimmune thyroiditis [34]. According to Jin *et al*., thyroid autoantibodies are present in 21.5% of patients with LADA, with 16.3% and 18.5% being positive for TGAb and TPOAb, respectively [35]. The prevalence of thyroid disease in patients with LADA is 17.7% [36], while patients with higher anti-GAD65 titer were more likely to exhibit thyroid autoimmunity [35] and have anti-TPO antibodies [37]. More precisely, middle and C-terminal epitopes of anti-GAD65 were correlated with higher anti-GAD titer, and greater risk of autoimmune thyroid disorders, in contrast with N-terminal epitopes [38]. In addition, positivity for more than one islet cell autoantibody increases the possibility of thyroid autoimmunity as a comorbidity [39]. Reversibly, in patients with HD, there is a predisposition for autoimmune DM, due to shared haplotypes [40]. The presence of anti-TPO antibodies may be interconnected with acceleration of autoimmune β-cell destruction [41]. Interestingly, hyperthyroidism as observed in hashitoxicosis has been shown to increase insulin clearance and subsequently magnify the risk of DKA [34].

In our patient, at least two autoimmune endocrine disorders coexist. This nosological entity is identified as polyglandular autoimmune syndrome (PAS) type III [42]. PAS has a strong genetic background, with many genes being actively involved in the immunological regulation and the activation of T cells in more than one endocrine structures at the same time. The antibodies produced may cross-react with more than one gland [34], leading to complex pathophysiological interconnections.

Chrousos highlighted that stress enhances the susceptibility to autoimmune disorders. Stress effectors affect both the thyroid hormone axis and metabolic systems [43], as chronic stress upregulates acute phase reactants, inducing metabolic disorders such as DM [44]. It should be assumed that the patient’s emotional stress along with the increased thyroid hormone titers can be eliciting factors for the rare presentation of DKA in a patient with LADA. Additionally, in DM and hyperthyroidism, there is increased activity of the hypothalamus–pituitary–adrenal axis [45]. Moreover, stressful situations, characterized by increased levels of cortisol, promote α-adrenergic signaling and decrease insulin secretion [46], subsequently leading to hyperglycemia. In addition, correlation of emotional stress with LADA has also been proposed [47]. HLA-DR4, among others, is found both in patients with HD and in those with stress-induced Graves’ disease, which indicates an indirect connection of HD and hashitoxicosis with stress [48].

We presented a remarkable case, characterized by simultaneous onset of two different autoimmune disorders: latent autoimmune diabetes in adults and Hashimoto’s disease, both presenting with rare manifestations, that is, diabetic ketoacidosis and hashitoxicosis. Owing to initial misdiagnosis and misleading findings, this particular case has revealed the necessity of a holistic diagnostic approach, including assessment of symptoms, complete physical examination, antibody tests, and understanding of pathophysiological correlations between the many different comorbidities.

Even if Glutamic acid decarboxylase antibody (anti-GAD) is the prominent autoantibody in LADA, other islet autoantibodies contribute significantly to the diagnosis of LADA and the assessment of β cell failure and intercurrent autoimmunity [49].

It has been disclosed that autoimmune disorders are multifactorial and interconnecting nosological entities with genetic and clinical heterogeneity, characteristics that make the diagnostic and therapeutic approach a challenging task. Subsequently, it is crucial, when an autoimmune disorder presents, to address and be familiar with all the comorbidities. More specifically, a conceivable theory is that pancreatic β-cell and thyroid autoimmunity coexist under a state of autoimmune susceptibility caused by loss of tolerance against self-antigens [50].

According to the American Diabetes Association, screening for thyroid and other autoimmunity is recommended for patients with T1DM [51]. Moreover, screening for thyroid antibodies and TSH is specifically advised in patients with LADA at onset and once every 2 years [35]. Boelaert *et al*. proposed screening for other autoimmune diseases in patients with autoimmune thyroid disease complaining about new or atypical symptoms [52].

However, screening might be unable to disclose and exclude extremely rare acute demonstrations of these disorders, highlighting the importance of the dexterity and awareness of the clinician.

## Data Availability

Authors confirm that all relevant data and materials are included in the manuscript and/or its supplementary information files.

## Abbreviations

LADA: Latent autoimmune diabetes in adults
T1DM: Type 1 diabetes mellitus
DM: Diabetes mellitus
Anti-GAD65: Autoantibodies against glutamic acid decarboxylase 65
GLP-1: Glucagon-like peptide
TSH: Thyroid-stimulating hormone
Anti-TG: Anti-thyroglobulin autoantibodies
Anti-TPO: Anti-thyroid peroxidase autoantibodies
DKA: Diabetic ketoacidosis
HD: Hashimoto’s disease
BMI: Body mass index
HbA1c: Glycosylated hemoglobin
FPG: Fasting plasma glucose
Anti-IA2: Anti-tyrosine phosphatase-related islet antigen 2
ICA: Anti-islet cell antibodies
IAA: Anti-insulin antibodies
PE: Physical examination
fT3: Free triiodothyronine
fT4: Free thyroxine
ZnT8A: Zinc transporter 8
PAS: Polyglandular autoimmune syndrome

## References

[1] Bluestone JA, Herold K, Eisenbarth G. Genetics, pathogenesis and clinical interventions in type 1 diabetes. Nature. 2010;464(7293):1293–300. 10.1038/nature08933. PMC4959889 20432533

[2] Naik RG, Palmer JP. Latent autoimmune diabetes in adults (LADA). Rev Endocr Metab Disord. 2003;4(3):233–41. 10.1023/a:1025148211587. 14501174

[3] Pozzilli P, Di Mario U. Autoimmune diabetes not requiring insulin at diagnosis (latent autoimmune diabetes of the adult): definition, characterization, and potential prevention. Diabetes Care. 2001;24(8):1460–7. 10.2337/diacare.24.8.1460. 11473087

[4] Buzzetti R, Tuomi T, Mauricio D, Pietropaolo M, Zhou Z, Pozzilli P, Leslie RD. Management of latent autoimmune diabetes in adults: a consensus statement from an international expert panel. Diabetes. 2020;69(10):2037–47. 10.2337/dbi20-0017. PMC7809717 32847960

[5] Cashen K, Petersen T. Diabetic ketoacidosis. Pediatr Rev. 2019;40(8):412–20. 10.1542/pir.2018-0231. 31371634

[6] Pozzilli P, Pieralice S. Latent autoimmune diabetes in adults: current status and new horizons. Endocrinol Metab. 2018;33(2):147–59. 10.3803/EnM.2018.33.2.147. PMC6021307 29947172

[7] Nadhem O, Nakhla E, Smalligan RD. Diabetic ketoacidosis as first presentation of latent autoimmune diabetes in adult. Case Rep Med. 2015;2015: 821397. 10.1155/2015/821397 (**Epub 2015 Mar 5**). PMC4365329 25834574

[8] Ray S, Sarkar D, Ganguly S, Maiti A. An unusual presentation of latent autoimmune diabetes in adults. Med J Malaysia. 2012;67(1):116–7. 22582562

[9] Raddatz V, Durruty P, Briones G, López G, Soto N, García de los Ríos M. Subtipos, “no clásicos” de diabetes mellitus [Non classical subtypes of diabetes mellitus]. Rev Med Chil. 2001;129(8):853–60 (**Spanish**). 11680958

[10] Caturegli P, De Remigis A, Rose NR. Hashimoto thyroiditis: clinical and diagnostic criteria. Autoimmun Rev. 2014;13(4–5):391–7. 10.1016/j.autrev.2014.01.007 (**Epub 2014 Jan 13**). 24434360

[11] Wasniewska M, Corrias A, Salerno M, Lombardo F, Aversa T, Mussa A, Capalbo D, De Luca F, Valenzise M. Outcomes of children with hashitoxicosis. Horm Res Paediatr. 2012;77(1):36–40. 10.1159/000334640 (**Epub 2012 Jan 26**). 22286076

[12] Litta Modignani R, Barantani E, Mazzolari M, Pincetti Nervi M, Macchi R. Chronic autoimmune thyroid disease. Ann Ital Med Int. 1991;6(4):420–6. 1804288

[13] Nabhan ZM, Kreher NC, Eugster EA. Hashitoxicosis in children: clinical features and natural history. J Pediatr. 2005;146(4):533–6. 10.1016/j.jpeds.2004.10.070. 15812459

[14] Soultati AS, Dourakis SP, Alexopoulou A, Deutsch M, Archimandritis AJ. Simultaneous development of diabetic ketoacidosis and Hashitoxicosis in a patient treated with PEGylated interferon-alpha for chronic hepatitis C. World J Gastroenterol. 2007;13(8):1292–4. 10.3748/wjg.v13.i8.1292. PMC4147013 17451219

[15] Laugesen E, Østergaard JA, Leslie RD, Danish Diabetes Academy Workshop and Workshop Speakers. Latent autoimmune diabetes of the adult: current knowledge and uncertainty. Diabet Med. 2015;32(7):843–52. 10.1111/dme.12700 (**Epub 2015 Feb 7. Erratum in: Diabet Med. 2015 Dec;32(12):1670**). PMC4676295 25601320

[16] Cervin C, Lyssenko V, Bakhtadze E, Lindholm E, Nilsson P, Tuomi T, Cilio CM, Groop L. Genetic similarities between latent autoimmune diabetes in adults, type 1 diabetes, and type 2 diabetes. Diabetes. 2008;57(5):1433–7. 10.2337/db07-0299 (**Epub 2008 Feb 29**). 18310307

[17] Lampasona V, Petrone A, Tiberti C, Capizzi M, Spoletini M, di Pietro S, Songini M, Bonicchio S, Giorgino F, Bonifacio E, Bosi E, Buzzetti R, Non Insulin Requiring Autoimmune Diabetes (NIRAD) Study Group. Non Insulin Requiring Autoimmune Diabetes (NIRAD) Study Group. Zinc transporter 8 antibodies complement GAD and IA-2 antibodies in the identification and characterization of adult-onset autoimmune diabetes: Non Insulin Requiring Autoimmune Diabetes (NIRAD) 4. Diabetes Care. 2010;33(1):104–8. 10.2337/dc08-2305 (**Epub 2009 Oct 6**). PMC2797953 19808923

[18] Hawa MI, Kolb H, Schloot N, Beyan H, Paschou SA, Buzzetti R, Mauricio D, De Leiva A, Yderstraede K, Beck-Neilsen H, Tuomilehto J, Sarti C, Thivolet C, Hadden D, Hunter S, Schernthaner G, Scherbaum WA, Williams R, Brophy S, Pozzilli P, Leslie RD; Action LADA consortium. Adult-onset autoimmune diabetes in Europe is prevalent with a broad clinical phenotype: Action LADA 7. Diabetes Care. 2013;36(4):908-13. 10.2337/dc12-0931. Epub 2012 Dec 17. Erratum in: Diabetes Care. 2014;37(5):1494. PMC3609504 23248199

[19] Fourlanos S, Perry C, Stein MS, Stankovich J, Harrison LC, Colman PG. A clinical screening tool identifies autoimmune diabetes in adults. Diabetes Care. 2006;29(5):970–5. 10.2337/diacare.295970. 16644622

[20] Krause S, Landherr U, Agardh C-D, Hausmann S, Link K, Hansen JM, Lynch KF, Powell M, Furmaniak J, Rees-Smith B, Bonifacio E, Ziegler AG, Lernmark Å, Achenbach P. GAD autoantibody affinity in adult patients with latent autoimmune diabetes, the study participants of a GAD65 vaccination trial. Diabetes Care. 2014;37(6):1675–80. 10.2337/dc13-1719. 24598244

[21] Radtke MA, Midthjell K, Nilsen TI, Grill V. Heterogeneity of patients with latent autoimmune diabetes in adults: linkage to autoimmunity is apparent only in those with perceived need for insulin treatment: results from the Nord-Trøndelag Health (HUNT) study. Diabetes Care. 2009;32(2):245–50. 10.2337/dc08-1468 (**Epub 2008 Nov 10**). PMC2628687 19001190

[22] Buzzetti R, Di Pietro S, Giaccari A, Petrone A, Locatelli M, Suraci C, Capizzi M, Arpi ML, Bazzigaluppi E, Dotta F, Bosi E, Non Insulin Requiring Autoimmune Diabetes Study Group. High titer of autoantibodies to GAD identifies a specific phenotype of adult-onset autoimmune diabetes. Diabetes Care. 2007;30(4):932–8. 10.2337/dc06-1696. 17392553

[23] Kasuga A, Maruyama T, Nakamoto S, Ozawa Y, Suzuki Y, Saruta T. High-titer autoantibodies against glutamic acid decarboxylase plus autoantibodies against insulin and IA-2 predicts insulin requirement in adult diabetic patients. J Autoimmun. 1999;12(2):131–5. 10.1006/jaut.1998.0261. 10047433

[24] American Diabetes Association. Pharmacologic approaches to glycemic treatment American Diabetes Association. Diabetes Care. 2017;40(Supplement 1):S64–74. 10.2337/dc17-S011. 27979895

[25] Omotosho YB, Ying GW, Stolar M, Mallari AJP. COVID-19-induced diabetic ketoacidosis in an adult with latent autoimmune diabetes. Cureus. 2021;13(1): e12690. 10.7759/cureus.12690. PMC7881111 33604221

[26] Rahmadi A, Decroli E, Kam A. Sepsis in latent autoimmune diabetes in adults with diabetic ketoacidosis: a case report. Open Access Maced J Med Sci. 2019;7(20):3501–4. 10.3889/oamjms.2019.687. PMC6980811 32002083

[27] Nabhan F, Emanuele MA, Emanuele N. Latent autoimmune diabetes of adulthood. Unique features that distinguish it from types 1 and 2. Postgrad Med. 2005;117(3):7–12. 10.3810/pgm.2005.03.1597. 15782668

[28] Vanderpump MP, Tunbridge WM, French JM, Appleton D, Bates D, Clark F, Grimley Evans J, Hasan DM, Rodgers H, Tunbridge F, *et al*. The incidence of thyroid disorders in the community: a twenty-year follow-up of the Whickham Survey. Clin Endocrinol. 1995;43(1):55–68. 10.1111/j.1365-2265.1995.tb01894.x. 7641412

[29] Benvenga S, Trimarchi F. Changed presentation of Hashimoto’s thyroiditis in North-Eastern Sicily and Calabria (Southern Italy) based on a 31-year experience. Thyroid. 2008;18(4):429–41. 10.1089/thy.2007.0234. 18399767

[30] Iddah MA, Macharia BN. Autoimmune thyroid disorders. ISRN Endocrinol. 2013;26(2013): 509764. 10.1155/2013/509764. PMC3710642 23878745

[31] Hashitoxicosis: an uncommon presentation of autoimmune thyroid BS Morris—Proceedings of UCLA Healthcare, 2013

[32] Williamson S, Greene SA. Incidence of thyrotoxicosis in childhood: a national population based study in the UK and Ireland. Clin Endocrinol. 2010;72(3):358–63. 10.1111/j.1365-2265.2009.03717.x (**Epub 2009 Sep 21**). 19769613

[33] Dunne C, De Luca F. Long-term follow-up of a child with autoimmune thyroiditis and recurrent hyperthyroidism in the absence of TSH receptor antibodies. Case Rep Endocrinol. 2014;2014: 749576. 10.1155/2014/749576 (**Epub 2014 Jul 9**). PMC4119923 25114812

[34] Biondi B, Kahaly GJ, Robertson RP. Thyroid dysfunction and diabetes mellitus: two closely associated disorders. Endocr Rev. 2019;40(3):789–824. 10.1210/er.2018-00163. PMC6507635 30649221

[35] Jin P, Huang G, Lin J, Yang L, Xiang B, Zhou W, Zhou Z. High titre of antiglutamic acid decarboxylase autoantibody is a strong predictor of the development of thyroid autoimmunity in patients with type 1 diabetes and latent autoimmune diabetes in adults. Clin Endocrinol. 2011;74(5):587–92. 10.1111/j.1365-2265.2011.03976.x. 21470281

[36] Peters KE, Chubb SAP, Bruce DG, Davis WA, Davis TME. Prevalence and incidence of thyroid dysfunction in type 1 diabetes, type 2 diabetes and latent autoimmune diabetes of adults: the Fremantle Diabetes Study Phase II. Clin Endocrinol. 2020;92(4):373–82. 10.1111/cen.14164 (**Epub 2020 Feb 3**). 31984536

[37] Zampetti S, Capizzi M, Spoletini M, Campagna G, Leto G, Cipolloni L, Tiberti C, Bosi E, Falorni A, Buzzetti R, NIRAD Study Group. GADA titer-related risk for organ-specific autoimmunity in LADA subjects subdivided according to gender (NIRAD study 6). J Clin Endocrinol Metab. 2012;97(10):3759–65. 10.1210/jc.2012-2037 (**Epub 2012 Aug 3**). 22865904

[38] Jin P, Huang G, Lin J, Luo S, Zhou Z. Epitope analysis of GAD65 autoantibodies in adult-onset type 1 diabetes and latent autoimmune diabetes in adults with thyroid autoimmunity. Acta Diabetol. 2011;48(2):149–55. 10.1007/s00592-010-0250-0 (**Epub 2011 Jan 7**). 21212992

[39] Lohmann T, Kellner K, Verlohren HJ, Krug J, Steindorf J, Scherbaum WA, Seissler J. Titre and combination of ICA and autoantibodies to glutamic acid decarboxylase discriminate two clinically distinct types of latent autoimmune diabetes in adults (LADA). Diabetologia. 2001;44(8):1005–10. 10.1007/s001250100602. 11484077

[40] Collin P, Kaukinen K, Välimäki M, Salmi J. Endocrinological disorders and celiac disease. Endocr Rev. 2002;23(4):464–83. 10.1210/er.2001-0035. 12202461

[41] Takeda H, Kawasaki E, Shimizu I, Konoue E, Fujiyama M, Murao S, Tanaka K, Mori K, Tarumi Y, Seto I, Fujii Y, Kato K, Kondo S, Takada Y, Kitsuki N, Kaino Y, Kida K, Hashimoto N, Yamane Y, Yamawaki T, Onuma H, Nishimiya T, Osawa H, Saito Y, Makino H, Ehime Study. Clinical, autoimmune, and genetic characteristics of adult-onset diabetic patients with GAD autoantibodies in Japan (Ehime Study). Diabetes Care. 2002;25(6):995–1001. 10.2337/diacare.25.6.995. 12032105

[42] Kahaly GJ, Frommer L. Polyglandular autoimmune syndromes. J Endocrinol Invest. 2018;41(1):91–8. 10.1007/s40618-017-0740-9 (**Epub 2017 Aug 17**). 28819917

[43] Chrousos GP. Stress and disorders of the stress system. Nat Rev Endocrinol. 2009;5(7):374–81. 10.1038/nrendo.2009.106 (**Epub 2009 Jun 2**). 19488073

[44] Black PH. The inflammatory response is an integral part of the stress response: Implications for atherosclerosis, insulin resistance, type II diabetes and metabolic syndrome X. Brain Behav Immun. 2003;17(5):350–64. 10.1016/s0889-1591(03)00048-5. 12946657

[45] Chrousos GP, Gold PW. The concepts of stress and stress system disorders. Overview of physical and behavioral homeostasis. JAMA. 1992;267(9):1244–52 (**Erratum in: JAMA 1992 Jul 8;268(2):200**). 1538563

[46] Surwit RS, Feinglos MN. Stress and autonomic nervous system in type II diabetes. A hypothesis. Diabetes Care. 1988;11(1):83–5. 10.2337/diacare.11.1.83. 3276481

[47] Chen YL, Qiao YC, Song XN, Ling W, Zhao HL, Zhang XX. Emotional exhaustion-induced latent autoimmune diabetes in adults in a young lady: a CARE-compliant case report. Medicine. 2017;96(20): e6915. 10.1097/MD.0000000000006915. PMC5440145 28514308

[48] Vita R, Cernaro V, Benvenga S. Stress-induced hashitoxicosis: case report and relative HLA serotype and genotype. Rev Assoc Med Bras. 2019;65(6):830–3. 10.1590/1806-9282.65.6.830. 31340312

[49] Xiang Y, Huang G, Zhu Y, Zuo X, Liu X, Feng Q, Li X, Yang T, Lu J, Shan Z, Liu J, Tian H, Ji Q, Zhu D, Ge J, Lin L, Chen L, Guo X, Zhao Z, Li Q, Weng J, Jia W, Liu Z, Ji L, Yang W, Leslie RD, Zhou Z, China National Diabetes and Metabolic Disorders Study Group. Identification of autoimmune type 1 diabetes and multiple organ-specific autoantibodies in adult-onset non-insulin-requiring diabetes in China: a population-based multicentre nationwide survey. Diabetes Obes Metab. 2019;21(4):893–902. 10.1111/dom.13595 (**Epub 2018 Dec 25**). 30471182

[50] Kucera P, Nováková D, Behanová M, Novak J, Tlaskalová-Hogenová H, Andel M. Gliadin, endomysial and thyroid antibodies in patients with latent autoimmune diabetes of adults (LADA). Clin Exp Immunol. 2003;133(1):139–43. 10.1046/j.1365-2249.2003.02205.x. PMC1808742 12823288

[51] Comprehensive Medical Evaluation and Assessment of Comorbidities. Standards of Medical Care in Diabetes—2021 American Diabetes Association. Diabetes Care. 2021;44(Supplement 1):S40–52. 10.2337/dc21-S004. 33298415

[52] Boelaert K, Newby PR, Simmonds MJ, Holder RL, Carr-Smith JD, Heward JM, Manji N, Allahabadia A, Armitage M, Chatterjee KV, Lazarus JH, Pearce SH, Vaidya B, Gough SC, Franklyn JA. Prevalence and relative risk of other autoimmune diseases in subjects with autoimmune thyroid disease. Am J Med. 2010;123(2):183.e1-9. 10.1016/j.amjmed.2009.06.030. 20103030

